# Short-Term Immune Responses of Gilthead Seabream (*Sparus aurata*) Juveniles against *Photobacterium damselae* subsp. *piscicida*

**DOI:** 10.3390/ijms23031561

**Published:** 2022-01-29

**Authors:** Paulo Santos, Diogo Peixoto, Inês Ferreira, Ricardo Passos, Pedro Pires, Marco Simões, Pedro Pousão-Ferreira, Teresa Baptista, Benjamín Costas

**Affiliations:** 1 CIIMAR, Centro Interdisciplinar de Investigação Marinha e Ambiental, Terminal de Cruzeiros do Porto de Leixões, Av. General Norton de Matos s/n, 4450-208 Matosinhos, Portugal; dpeixoto@ciimar.up.pt (D.P.); ines.ferreira@ciimar.up.pt (I.F.); 2 ICBAS, Instituto de Ciências Biomédicas Abel Salazar, Universidade do Porto, Rua de Jorge Viterbo Ferreira, 4050-313 Porto, Portugal; 3 MARE, Centro de Ciências do Mar e do Ambiente, Instituto Politécnico de Leiria, Edifício CETEMARES, Av. Porto de Pesca, 2520-620 Peniche, Portugal; ricardo.passos@ipleiria.pt (R.P.); pedro.pires@ipleiria.pt (P.P.); marco.a.simoes@ipleiria.pt (M.S.); teresa.baptista@ipleiria.pt (T.B.); 4 IBMC, Instituto de Biologia Molecular e Celular, Universidade do Porto, Rua Alfredo Allen, 208, 4200-135 Porto, Portugal; 5 i3S, Instituto de Investigação e Inovação em Saúde, Universidade do Porto, Rua Alfredo Allen, 208, 4200-135 Porto, Portugal; 6 IPMA, Instituto Português do Mar e da Atmosfera, Parque Natural da Ria Formosa s/n, 8700-194 Olhao, Portugal; pedro.pousao@ipma.pt

**Keywords:** photobacteriosis, *Sparus aurata*, fish immunology, infection, red blood cells, neutrophils, Interleukin-34, haptoglobin

## Abstract

Photobacteriosis is a septicaemic bacterial disease affecting several marine species around the globe, resulting in significant economic losses. Although many studies have been performed related to the pathogen virulence and resistance factors, information regarding the host defence mechanisms activated once an infection takes place is still scarce. The present study was designed to understand innate immune responses of farmed juvenile gilthead seabream (*Sparus aurata*) after *Photobacterium damselae* subsp. *piscicida* (*Phdp*) infection. Therefore, two groups of seabream juveniles were intraperitoneally injected with 100 µL of PBS (placebo) or 100 µL of exponentially growing *Phdp* (1 × 10^6^ CFU/mL; infected). The blood, plasma, liver, and head kidney of six fish from each treatment were sampled immediately before infection and 3, 6, 9, 24 and 48 h after infection for the broad screening of fish immune and oxidative stress responses. Infected animals presented marked anaemia, neutrophilia and monocytosis, conditions that are correlated with an increased expression of genes related to inflammation and phagocytic activity. Similar studies with different fish species and bacteria can be useful for the definition of health biomarkers that might help fish farmers to prevent the occurrence of such diseases.

## 1. Introduction

One of the biggest challenges related to fish production intensification is related to the increasing occurrence of pathological threats that lead to relevant monetary losses. Among them, photobacteriosis or fish pasteurellosis presents as a septicemic disease caused by *Photobacterium damselae* subsp. *piscicida (Phdp)*, a Gram-negative facultative intracellular halophilic bacterium [1]. The first report of this infection occurred in a wild population of white perch in 1963 [2], and the first isolation in Mediterranean countries occurred at the beginning of the 1990s in a Spanish gilthead seabream fish farm [3]. This disease can affect a wide diversity of marine species, including seabream, seabass, salmon [4], sole (*Solea senegalensis* and *Solea Solea*) [5], meagre (*Argyrosomus regius*) [6], yellowtail (*Seriola quinqueradiata*) and cobia (*Rachycentron canadum*) [1].

Photobacteriosis incidence increases during warm periods, and pathology progression has been associated with temperatures above 18–20 °C [7], low salinity, and poor water quality [4]. The susceptibility of gilthead seabream to *Phdp* varies with fish development, with larvae and juveniles being less resistant to infection (with mortalities reaching 90–100%), whereas fish over 50 g in weight present more resilience due to phagocytosis [5]. In Portugal, the last report regarding infection of gilthead seabream fingerlings with *Phdp* resulted in mortalities reaching 11% a day, affecting all animals from a fish farm facility [8].

Infection usually takes place without clinical external lesions, with mild haemorrhagic regions in the head and gills [4,8], anorexia and dark skin [7] sometimes being observable. On the other hand, infected fish usually present internal signs of disease such multifocal necrosis in the liver, spleen, and kidney [1], and in some cases a pale liver [8], splenomegaly and kidney enlargement [6]. It is also common to find whitish granulomatous nodules on the kidney and spleen with diameters varying between 0.5 and 3.5 mm [9].

The infectious process is complex and depends on the bacterial ability to avoid the host defence cells. Although the pathogenesis is still not fully understood, especially the invasion of non-phagocytic cells, several virulence factors are considered to increase *Phdp* resistance and proliferation. The polysaccharide composition of the capsule confers protection to bacteria against bactericidal serum activity, thus enhancing pathogen survival [10]. Another strategy that enables the bacteria to resist therapeutic measures is the ability to survive on host phagocytic cells [1]. The acquisition of iron from the host is also very important for bacterial survival and proliferation, and *Phdp* contains high-affinity iron-binding siderophores that allow the uptake of this metal from heme compounds of host erythrocytes [11]. This bacterium can also release extracellular products with haemolytic and phospholipase activities which can produce host cells damage and disruption [12], and an apoptotic inducing protein with 56 KDa (AIP56) was discovered to be present on virulent strains of *Phdp* resulting in phagocytic cell apoptosis on European seabass [13].

Several diagnostic tools have also been developed over the last three decades for the correct identification of the disease. However, the genetic similarities between *Photobacterium damselae* subsp. *damselae* and *Phdp* revealed out a problem which was only overcome by molecular approaches requiring more than one step, such as Multiplex PCR [14,15] or the amplification of the capsular polysaccharide gene (CPS) with an additional culture step on thiosulfate-citrate-bile salts-sucrose (TCBS) [16].

Even though treatment measures have been widely used to combat photobacteriosis, the recent concerns about antibiotics resistance, environmental pollution and animal and human health contributed to the development of vaccines. This prophylactic measure’s effectiveness differs with fish species, size and vaccine formulation [17], and since *Phdp* frequently affects seabream juveniles weighing from 10 to 30 g, conventional vaccines consisted of inactivated products resultant from heat- or formalin-killed bacteria, which were administered by dipping fish on early larval stages (1–2 g) [18]. In 2016, the veterinary pharmaceutical company HIPRA (Girona, Spain) was permitted to commercialize a vaccine, ICTHIOVAC^®^PD, specifically formulated for juvenile seabream, conferring 5 months of protection after dip administration of two inactivated Phdp strains for fish with 1 to 2 g [19]. Depending on fish rearing facilities and prophylactic strategies, fish might be revaccinated through intraperitoneal administration, and boosters usually occur when fish reach 15 to 20 g.

Previous studies in which gilthead seabream were challenged with *Phdp* resulted in enhanced immune activity, more specifically plasmatic antiproteases [20] and the expression of genes related to inflammation and macrophage activation and differentiation [5].

Since information concerning host response against photobacteriosis is scarce, the aim of this study is to evaluate the immune modulation of gilthead seabream juveniles when infected with *Phdp*. Data from this study might be useful for the development of early detection methods which can help fish farmers to prevent the presence and development of photobacteriosis.

## 2. Results

### 2.1. Bacterial Challenge

**Figure 1:**
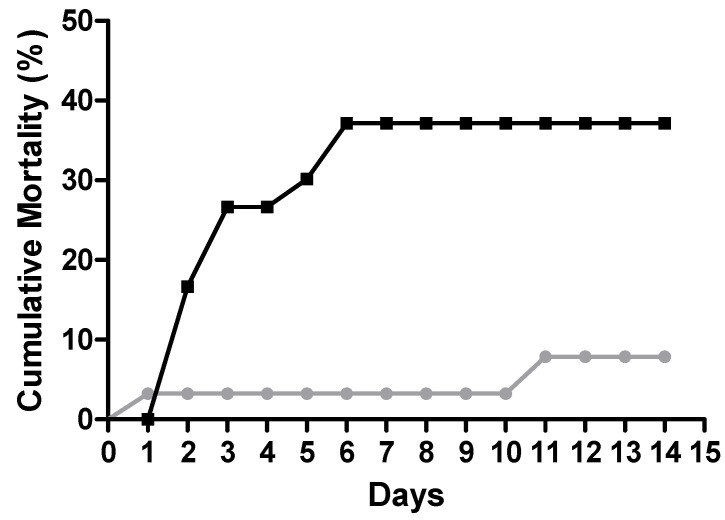
Cumulative mortality (%) of gilthead seabream after PBS (●) or Phdp (■) intraperitoneal injection (*n* = 60).

Evaluating the effect of bacterial infection on fish survival for 14 days within different treated groups (*n* = 60), Figure 1 presents a clear and marked difference (X^2^ = 0.0053) between fish that were inoculated with Phdp (cumulative mortality of 36.7%) and fish injected with PBS (cumulative mortality of 6.7%). It is also possible to observe that fish that died due to bacterial infection were only found during the first 6 days of the disease, this being the normal death timing of this bacterial disease.

### 2.2. Haematological Analysis

Blood from 12 fish was sampled before the challenge (Time 0), while six fish were examined 3, 6, 9, 24 and 48 h post-injection.

**Figure 2:**
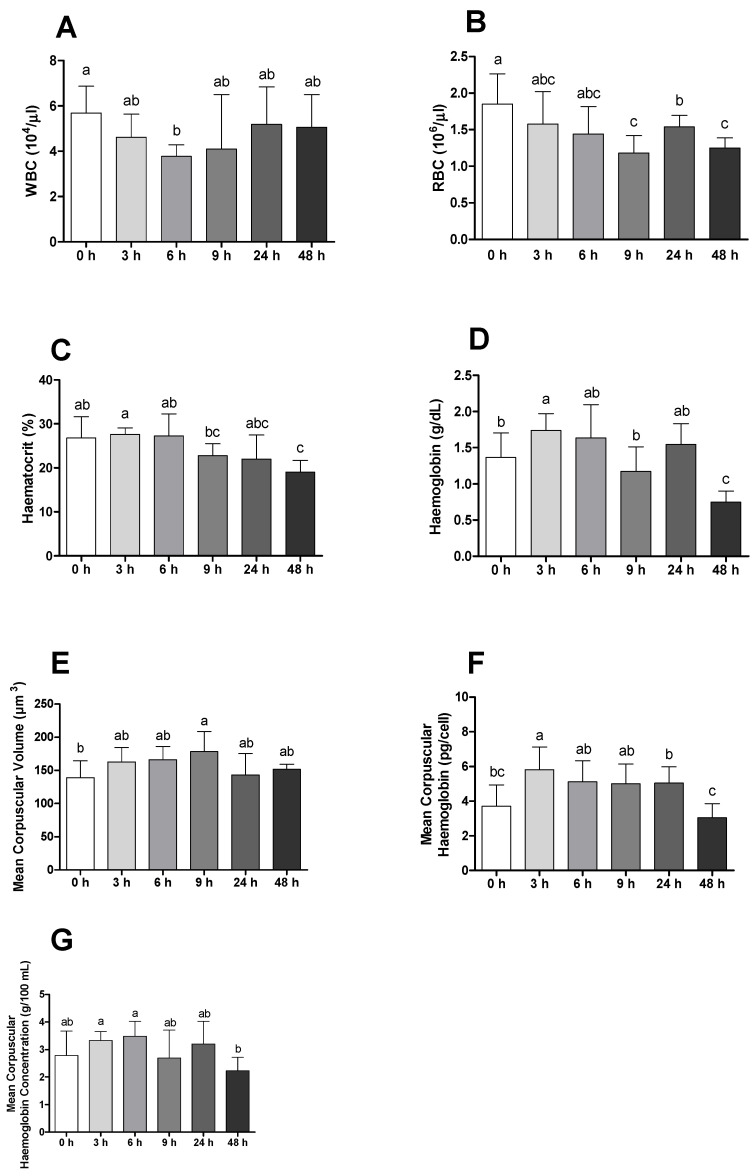
(**A**) White blood cells (WBC, ×10^4^/µL), (**B**) red blood cells (RBC, ×10^6^/µL), (**C**) haematocrit (Ht, %), (**D**) haemoglobin (Hg, g/dL), (**E**) mean corpuscular volume (MCV µm^3^), (**F**) mean corpuscular haemoglobin (MCH, pg/cell) and (**G**) mean corpuscular haemoglobin concentration (MCHC, g/100 mL) of gilthead seabream before and after bacterial challenge. Data are expressed as means ± SD (*n* = 12 for control animals and *n* = 6 on time course animals). Different lowercase letters stand for significant differences among different times between control and infected animals. (*t*-student test or Kruskal–Wallis; *p* ≤ 0.05).

Regarding haematological parameters, reduced values on white and red blood cells were observed in infected animals when compared with animals before bacterial insult, and this difference is clearly observable after 6 and 48 h, respectively (Figure 2A,B). Additionally, haematocrit and haemoglobin values presented a similar trend, with significant differences being registered 48 h post-infection (Figure 2C,D). Differences between infected and placebo animals on the same sampling times are also observable on these parameters, with infected animals presenting lower values after 48 h (Table S1). Although haematological ratios suffered from small discrepancies over time, especially within the first 6 h, it is possible to observe that all parameters returned to normal values 48 h after challenge (Figure 2E–G).

**Figure 3:**
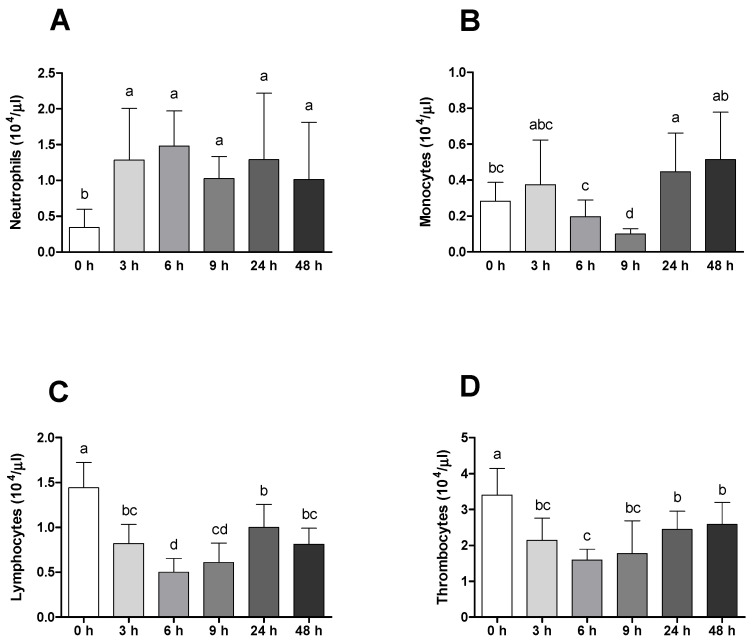
Absolute values (×10^4^/µL) of peripheral blood leukocytes ((**A**) neutrophils, (**B**) monocytes, (**C**) lymphocytes and (**D**) thrombocytes) of gilthead seabream before and after bacterial challenge. Data are expressed as means ± SD (*n* = 12 for control animals and *n* = 6 on time course animals). Different lowercase letters stand for significant differences among different times between control and infected animals. (*t*-student test or Kruskal–Wallis; *p* ≤ 0.05).

A significant variation in the peripheral leucocyte population on blood smears was registered. Neutrophils increased in infected animals at all times compared to undisturbed fish (Figure 3A). It is also possible to observe that the neutrophils population returned to basal values 24 h after injection in the placebo group (Table S2). Peripheral monocytes decreased after 9 h followed by monocytosis at 24 and 48 h in infected animals (Figure 3B). Lymphocytes and thrombocytes varied in a similar way, with total values decreasing after 6 h with a later increase 24 and 48 h post-infection (Figure 3C,D). Still focusing on the last two parameters, infected animals presented lower values when compared with sham ones after 6 and 9 h of IP (Table S2).

### 2.3. Innate Humoral Parameters

**Figure 4:**
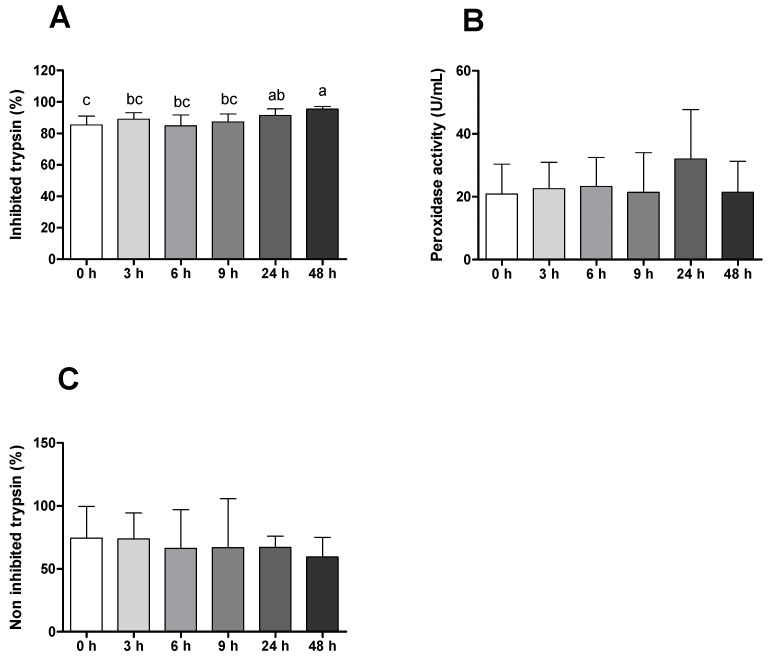
(**A**) Plasma antiprotease (%), (**B**) peroxidase (units/mL) and (**C**) proteases activities (%) of gilthead seabream before and after bacterial challenge. Data are expressed as means ± SD (*n* = 12 for control animals and *n* = 6 on time course animals). Different lowercase letters stand for significant differences among different times between control and infected animals. (*t*-student test or Kruskal–Wallis; *p* ≤ 0.05).

Plasma antiproteases activity increased during infection with the highest values at 48 h after infection (Figure 4A). Regarding peroxidase, it is possible to see an increase in the activity 24 h after infection (Figure 4B), while plasma proteases tended to decrease their activity along the infection period (Figure 4C). Interesting findings were also observed in plasma peroxidase and antiprotease activities comparing infected with placebo groups, with a significant increase in this activity in fish injected with *Phdp* 6 and 48 h after challenge, respectively (Table S3).

### 2.4. Oxidative Stress

**Figure 5:**
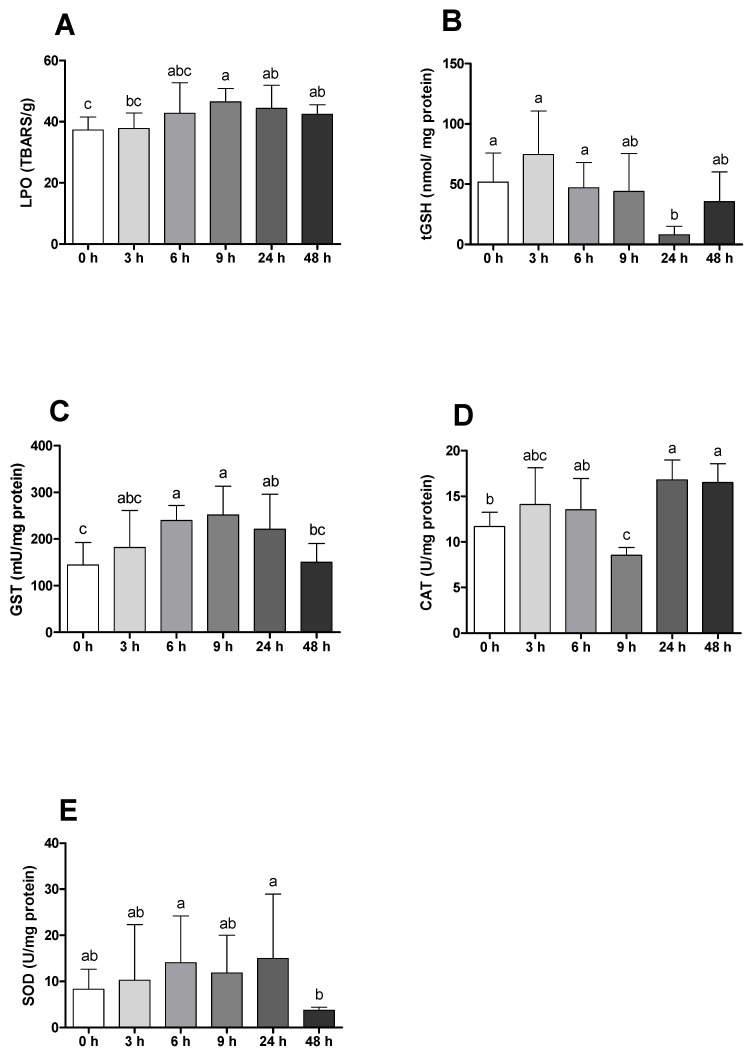
Oxidative stress parameters of gilthead seabream before and after bacterial challenge. (**A**) Lipid peroxidation (LPO, TBARS/g); (**B**) total glutathione (tGSH, nmol/mg protein); (**C**) glutathione S-transferase activity (GST, mU/mg protein); (**D**) catalase activity (CAT, U/mg protein); (**E**) superoxide dismutase activity (SOD, U/mg protein). Data are expressed as means ± SD (*n* = 12 for control animals and *n* = 6 on time course animals). Different lowercase letters stand for significant differences among different times between control and infected animals. (*t*-student test or Kruskal–Wallis; *p* ≤ 0.05).

Gilthead seabream juveniles presented higher levels of LPO after 9 h of bacterial challenge (Figure 5A). Regarding tGSH, a decrease can be observed 24 h post-infection with a further recovery of basal levels (Figure 5B). GST and CAT varied in opposite senses, with GST increasing until 9 h and CAT decreasing over the same sampling time (Figure 5C,D). SOD followed a constant increase until 24 h, and after that time suffered from a significant decrease (Figure 5E). Differences between placebo and infected animals were found in LPO and CAT activity, with these parameters being increased in infected fish after 48 and 24 h, respectively, while tGSH registered lower values than the sham group 24 h post-injection (Table S4).

### 2.5. Gene Expression Analysis

**Figure 6:**
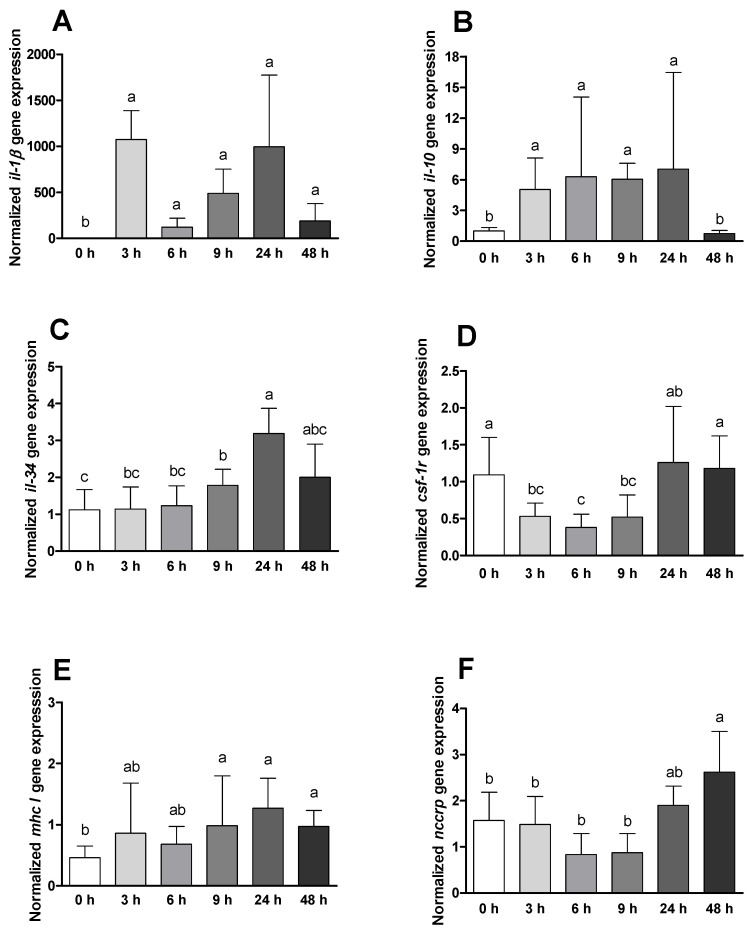
Head-kidney quantitative expression of (**A**) interleukin-1β (*il-1 β*), (**B**) interleukin-10 (*il-10*), (**C**) interleukin-34 (*il-34*), (**D**) colony stimulation factor 1 receptor (*csf1-r*), (**E**) major histocompatibility complex I (mhc I) and (**F**) non-specific cytotoxic cell receptor protein (*nccrp*) of gilthead seabream before and after bacterial challenge. Data are expressed as means ± SD (*n* = 12 for control animals and *n* = 6 on time course animals). Different lowercase letters stand for significant differences among different times between control and infected animals (*t*-student test or Kruskal–Wallis; *p* ≤ 0.05).

The mRNA expression of *il1-β* and *il-10* was found to increase in infected animals immediately after infection and were maintained along sampling times (Figure 6A,B). Similar differences were observed regarding these genes when comparing infected with placebo animals, with a significant increase for challenged animals at times 3, 9 and 24 (Table S5). IL-34 expression increased over time with a peak at 24 h post *Phdp* insult (Figure 6C). On the other hand, *csf-1r* levels were decreased within the first 9 h of infection, with a later increase (Figure 6D). Differences between different treatments at the same sampling points on the expression of *il-34* and *csf-1r* resulted in augmented values on fish injected with bacteria especially 48 h after infection (Table S5). *mhc I* and *nccrp* were found to be upregulated after 9 and 48 h of infection, respectively (Figure 6E,F).

**Figure 7:**
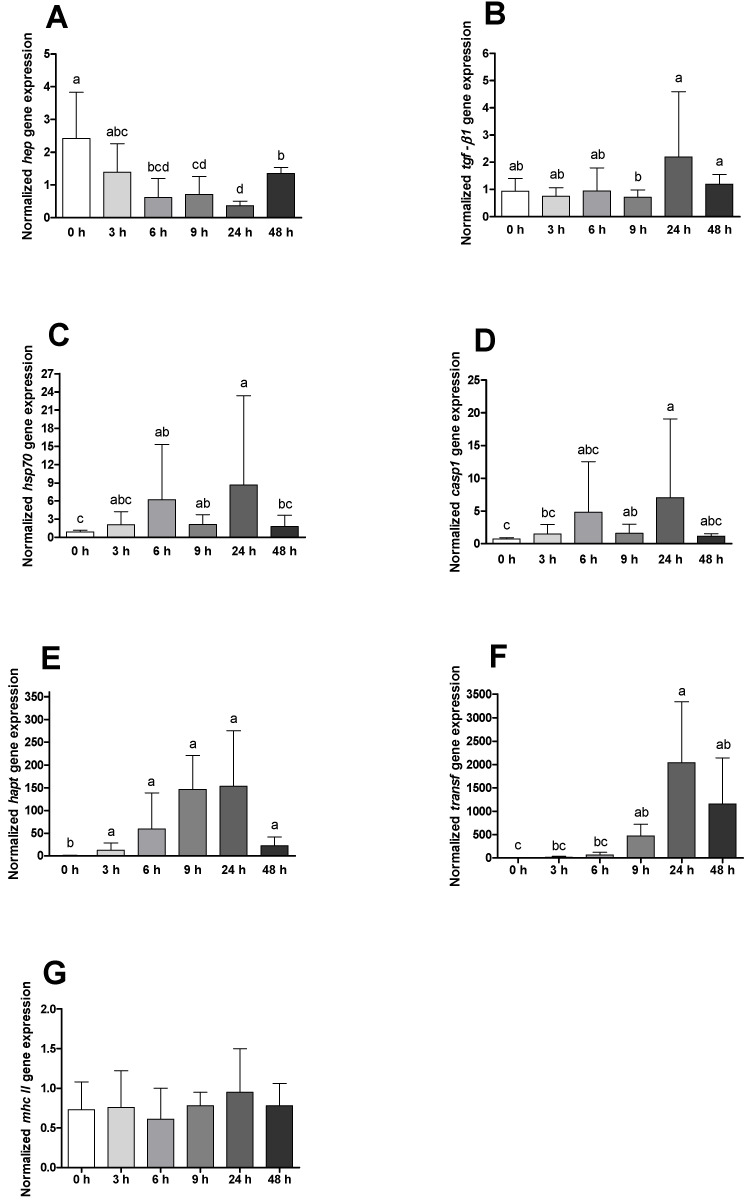
Head-kidney quantitative expression of (**A**) hepcidin (*hep*), (**B**) transforming growth factor β1 (tfg-*β1*), (**C**) heat shock protein 70 (hsp70), (**D**) caspase 1 (*casp1*), (**E**) haptoglobin (*hapt*), (**F**) transferrin (*transf*) and (**G**) major histocompatibility complex II (*mhc II*) of gilthead seabream before and after bacterial challenge. Data are expressed as means ± SD (*n* = 12 for control animals and *n* = 6 on time course animals). Different lowercase letters stand for significant differences among different times between control and infected animals (*t*-student test or Kruskal–Wallis; *p* ≤ 0.05).

The mRNA expression of *hep* was found to be decreased from the first hours of the disease onwards (Figure 7A), while *tgf-β1*, *hsp70*, *casp1*, *hapt* and *transf* increased with peaks observed 24 h post-challenge (Figure 7B–F). No significant differences were found on *mhc II* transcripts (Figure 7G). Comparing sham-injected animals and infected ones, is observable that real infection led to augmented expression of *mhc II*, *hep*, *hsp70* and *casp1* after 24 h, while for *hapt* and *transf*, this difference was observed one sampling time earlier (9 h). A later but similar response was found in the expression of *tgf-β1*, which presented higher relative expression values in infected animals 48 h post-infection (Table S5).

## 3. Discussion

The modulation of fish innate immune machinery after an intraperitoneal bacterial insult was here studied. Even though there are several available studies evaluating teleost defence mechanisms in response to infection with *Phdp* [21,22,23,24,25,26,27], the present approach provides a wider and more complete analysis of the mechanisms activated in response to this pathogen.

Evaluation of mortality rates resulted in a higher death percentage in infected fish. In addition, cumulative mortality was similar to that observed in studies performed on seabass [23] and Senegalese sole [25], using the same route (i.p) and approximated doses. Furthermore, infected fish started to die within the first 6 days after pathogen inoculation (similarly to natural acute infection), and pathogen presence was confirmed by bacterial isolation on TSA plates, with infected animals exhibiting mild liver and spleen enlargement. Mortality in control groups was low and most likely related to the stressful situation imposed due to handling and PBS injection, since no external or internal disease signs were detected.

Regarding fish haematological data, it is possible to observe that fish challenged with bacteria presented an anaemic condition when compared with unchallenged ones, with erythrocyte lysis due to bacterial enzyme or toxic action being a possible explanation for this [12,28]. Additionally, haematocrit presented lower values in infected animals 48 h after bacterial stimulus, with this finding being consistent with that already observed in seabass and meagre [21,29]. A possible strategy for the proliferation of *Phdp* in the host was hypothesized by [11], since bacteria grown with added haemin and haemoglobin presented higher virulence. Moreover, *Phdp* extracted from infected Senegalese sole showed an increased expression of genes involved in pathogen iron acquisition, such as iron regulatory proteins 1 and 2 (with active roles on the synthesis of siderophore piscidin) and HutB and HutD (encoding for hemic binding protein) [30]. Compiling all data, these blood parameters represent a good opportunity for the establishment of disease biomarkers due to its easy and fast evaluation.

Differential peripheral leucocytes were shown to be significantly influenced by *Phdp*. The results are in line with previous reports that showed clear neutrophilia, monocytosis [27,31,32] and lymphopenia [22,33] in infected animals within the first 24 h of infection. In addition, infected animals also presented reduced thrombocyte values, a result that, although not being very common during photobacteriosis, has been described during infection episodes with other Gram-negative bacterial species [34], supporting the hypothesis that these cells may have the ability to migrate to the inflammatory focus to cope with pathogen invasion. Moreover, sham-injected seabream also demonstrated an activated innate immune response to the stimulus, which reinforces the importance of having good control treatments for a better understanding of host/pathogen interactions. The slight increase in plasma peroxidase and antiprotease activities observed in infected animals 24 h after infection is in line with other findings from European seabass infection [35,36]. It is also plausible that these slight differences were not so clearly seen between control and infected groups due to neutrophil degranulation on the peritoneal cavity, thus decreasing its concentration in plasma. Another explanation for this finding is correlated with the action of the bacterial toxin AIP-56 on phagocytic cells [13], inducing selective apoptotic destruction of macrophages and neutrophils, culminating in reduced pathogen clearance and antimicrobial products release [37].

Since the liver plays a key role in detoxification by removing reactive oxygen species (ROS), the activity of antioxidant enzymes was also studied. Superoxide dismutase (SOD) is known to catalyse the reaction that leads to the production of hydrogen peroxide and oxygen by stabilization of superoxide anion [38]. Catalase (CAT) is responsible for decomposing hydrogen peroxide into oxygen and water [39], contributing to the protection of cells to stress damage. The results from this study show that these two enzymes increased until 24 h in response to bacterial infection. Similarly, SOD and CAT have been found to be augmented in the liver of vaccinated rainbow trout against furunculosis [40], reinforcing their importance in fish oxidative radical clearance. The increase in lipid peroxidation (LPO) levels since the first hours of disease development was also remarkable. This result was usually found in experiments with finfish infected with Gram-negative bacteria [41,42], suggesting that the rate of hepatic production of ROS during infection can produce a devastating effect on cell membranes. Additionally, glutathione S-transferase takes part in the metabolism of endogenous substances. The increase in this phase II detoxifying enzyme is associated with an enhanced oxidative stress resistance [41], and data from the present study support the idea that GST can have a protective role against *Phdp* in the first hours after infection. The fact that total glutathione was found to decrease in infected animals further suggests that it was consumed by GST.

The modulatory effect of bacterial challenge on the expression of pro- and anti-inflammatory genes has provided good insights about the mechanisms involved in fighting this disease. In the present study, the observed increase in *il-1β* expression from infected gilthead seabream was in line with that already observed in teleosts submitted to bacterial diseases. In fact, *il-1β* expression evaluation after a bacterial challenge is a common approach, and similar studies have been performed [24,43,44,45] resulting in analogous variations. Indeed, IL-1β is a pro-inflammatory cytokine with a key role in the first stages of inflammation by attracting fish leucocytes [46]. *Caspase 1* expression levels followed a similar trend compared to *il-1β* variation over time (although with less greatness), since this inflammatory caspase’s function is to cleave and activate IL-1β, IL-18 and IL-33 [47]. This cleavage occurs at a phylogenetically conserved aspartate residue in seabass [48], and correlation between the concentration of both molecules has been described in the past using Senegalese sole as the infected host [26], supporting the hypothesis that this might be a preferred inflammatory pathway in gilthead seabream against *Phdp*. Notwithstanding, the perceived acute decrease in IL-1β after the 6 h could be the result of the activity of Phdp-related toxins, such as AIP56. The referred toxin exerts immune-inhibitory actions by cleaving the transcriptional factor NF-κB [49] and concomitant transcription of key pro-inflammatory cytokines, as the IL-1β. Nonetheless, a later recovery from host immune machinery was observed 24 h after challenge.

In order to maintain homeostasis during infection episodes, anti-inflammatory signals are also released. IL-10 is an anti-inflammatory cytokine produced by a high variety of immune cells and takes a pivotal role during inflammatory responses due to its ability to inhibit macrophages and monocytes, leading to decreased pro-inflammatory cytokines release, phagocytosis and host cells damage [50]. Besides that, IL-10 can also enhance the activation and proliferation of all kinds of lymphocytes. In the present study, photobacteriosis modulated this anti-inflammatory cytokine by augmenting its expression in a fast response. In this sense, our results are congruent with the literature [5,27,51,52], reinforcing its high importance in the control of inflammation.

As heat shock proteins (HSPs) were described previously as important chaperones involved in the initial stages of the inflammatory process after bacterial infection [53], the present study also focused in the expression levels of *hsp70*. Even though an augmented expression for *hsp70* was expected under a stressful stimulus, no significant differences were found on previous works after infections with *Phdp* in seabream and Senegalese sole [23,25]. Results from the present study are in agreement with the above cited works, and it is here hypothesized that this phenomenon might be triggered with apoptotic stimulation of phagocytes by bacteria, with consequent decreased inflammatory pathway activation.

Another important cellular population contributing to the fast elimination of pathogens is the so-called cytotoxic cells. NCCRP is a receptor protein expressed on non-specific cytotoxic cells that are intimately related to the inflammatory response [25]. The results from this study show an upregulation of this gene in infected animals 48 h after infection. Moreover, an increase in the mRNA expression of *mhc I* at 24 h following infection was also observed, suggesting that MHCI/CD8+ interaction could be another host strategy used to debelate *Phdp* infection. On the other hand, the expression of *mhc II* remained stable over time among both treatment groups, and since this molecule is present mainly after inflammatory signals on monocytes, macrophages and dendritic cells [54], there is a possibility that its expression might not be reached due to phagocytic cell apoptosis induced by AIP56. This hypothesis could also be related to the lack of changes observed in *tgf-β1* mRNA expression levels, which are also in line with that found in cobia and European seabass [36,55]. TGF-β is a multipotent cytokine affecting cell differentiation, proliferation, apoptosis and matrix production [56].

Data from the present study also showed an increase in the expression of *il-34* and *csf-1r* in infected fish at 48 h. IL-34 is a cytokine that has only recently been described in fish. This cytokine, together with CSF-1, has the capacity to bind to CSF-1r, resulting in the differentiation, proliferation and survival of monocytes, macrophages and osteoclasts [57,58]. Both CSF-1 and IL-34 indistinctly bind the receptor, even though variations in the macrophages’ secretome obtained by molecule ligation of either were detected [59]. Therefore, it could be hypothesized that both *il-34* and *csf-1r* transcripts seem to play a key role in gilthead seabream survival against *Phdp* by improving macrophage differentiation at 48 h after infection with *Phdp*. In fact, these data seem to be correlated with the increased level of circulating monocytes from infected fish at this time.

Hepcidin is an antimicrobial peptide (AMP) that also contributes to iron homeostasis by inhibiting cellular iron efflux from enterocytes, hepatocytes and macrophages through a mechanism that involves ferroportin cell internalization [60]. Since hepcidin is easily stimulated by pro-inflammatory cytokines, it was expected that the expression of this gene would increase drastically with the inflammatory response. However, no differences were found in the present study, and similar results were provided in a study with iron-deficient European seabass [61]. Furthermore, the acute anaemic state has been investigated in mice and related to decreased hepcidin gene expression [62].

Defensins are widely studied AMPs with multiple actions on the infectious process. Adding to its antimicrobial role, β-defensins are also involved in chemotactic tasks by attracting monocytes, T lymphocytes and immature dendritic cells as well as promoters of dendritic cell’s maturation and differentiation [63]. Since *β-defensin* mRNA transcription was not significantly affected by *Phdp* infection, it is not possible to affirm that this AMP enhances immune status during photobacteriosis episodes.

Past studies recognized the importance of haptoglobin and transferrin as molecules produced by fish that can chelate and recycle iron, thus decreasing the quantity of iron available for bacterial uptake [26]. In the present study, both genes presented high expression levels of mRNA, which make us believe that this might be a strategy to avoid the progression of *Phdp*. Furthermore, haptoglobin is recognized to be an important marker for stress [64], being a possible candidate for a farmed fish health biomarker.

## 4. Materials and Methods

### 4.1. Experimental Design

The current study was conducted under the supervision of accredited researchers in laboratory animal science by the Portuguese Veterinary Authority following FELASA category C recommendations. This experiment was performed according to the guidelines on the protection of animals used for scientific purposes (European Union directive 2010/63/EU).

Gilthead seabream juveniles were transferred from Estação Piloto de Piscicultura de Olhão (Olhão, Portugal) to Politécnico de Leiria facilities (CETEMARES, Leiria, Portugal), and quarantined for a period of 90 days. After this period, 132 fish (9.8 ± 2.2 g) were individually weighed and randomly distributed into 6 recirculating tanks of 60 L of seawater (*n* = 22, animal initial density = 19.6 Kg/m^3^, photoperiod 12 h light/12 h dark). The physicochemical parameters such as oxygen saturation (6.62 ± 0.04 mg/L), salinity (30.95 ± 0.06) and pH (8.04 ± 0.05) were monitored on a daily basis. Both temperature and ammonium/nitrite levels were kept constant throughout the trial (T = 25 ± 1 °C; NH_4_ and NO_2_, respectively, under 0.33 and 1.61 mg/L).

### 4.2. Bacterial Challenge

*Photobacterium damselae* subsp. *piscicida* (AQP17.1), kindly provided by Professor Alicia E. Toranzo (Departamento de Microbiologia y Parasitologia, Facultad de Biologia, Universidade de Santiago de Compostela, Santiago, Spain), was cultured on Erlenmeyer flasks containing 50 mL of TSB (1.5% of NaCl) (Difco Laboratories, New Jersey, USA and grown under continuous agitation (25 °C) for 48 h. After that, the contents of the flasks were transferred to 50 mL falcon tubes and centrifuged for 30 min at 3500 rpm. The supernatants of the centrifuged tubes were then discarded, and the remaining pellet was dissolved in phosphate buffered saline (PBS, GIBCO). Bacterial concentration was read at 600 nm and adjusted to 1 × 10^6^ CFU/mL. Half of the individuals were infected through peritoneal injection with 100 µL of the above suspension (1 × 10^5^ CFU/fish), while the other half of the individuals were kept as the control group and injected with the same volume of PBS. Infection was followed for 14 days, and animals that died during this period were registered in order to obtain mortality rates.

### 4.3. Sampling

Both infected and control groups were sampled immediately before infection (Time 0), and then 3, 6, 9, 24 and 48 h after the challenge. Two fish per tank were randomly sampled for each time point (*n* = 6 for treatment) and euthanized using 2-phenoxyethanol (0.5 mL/L). Blood samples were collected from the caudal vein using 1 mL syringes (previously prepared with 3000 units/mL of heparin). Blood samples were then placed in 1.5 mL heparinized tubes and gently homogenized for haematological analysis as described below. The remaining blood was centrifuged for 10 min at 10,000× *g* at 4 °C, and afterwards, plasma was collected and stored at −80 °C. Head-kidneys and livers were also aseptically collected for gene expression and oxidative stress analysis. After collection, head-kidneys were stored in RNA later (with a proportion of 1/10 *w*/*v*) at 4 °C for the first 24 h and then stored at −80 °C, and the livers were immediately frozen and stored at −80 °C.

### 4.4. Haematological Analyses and Blood Smears

Before centrifugation of homogenized blood, a small aliquot was reaped for white blood cells (WBC) and red blood cells (RBC) counts, haematocrit (Ht) and haemoglobin determination (Hb, SPINREACT kit, ref. 1001230, Girona, Spain). Mean corpuscular volume (MCV), mean corpuscular haemoglobin (MCH) and mean corpuscular haemoglobin concentration (MHCH) were also calculated:

MCV (mm^3^) = (Ht/RBC) × 10

MCH (pg/cell) = (Hb/RBC) × 10

MHCH (g/100 mL) = (Hb/Ht) × 100

The smears from heparinized blood were run through a single blood droplet and air-dried. After drying, the slides were fixed with a solution of formaldehyde-ethanol (90% absolute ethanol to 10% of 37% formaldehyde) for one minute [65]. Neutrophils were then marked for the detection of peroxidase activity, following a protocol described by [66]. Subsequently, slides were stained with Wright’s stain (Haemacolor, Merck) and observed under oil immersion (1000×). Leucocytes were identified and a differential count of neutrophils, monocytes, lymphocytes and thrombocytes was made in a total of 200 cells/smear. Relative counts were further converted for absolute values (×10^4^/mL) of each cell type using WBC results.

### 4.5. Innate Humoral Parameters

#### 4.5.1. Peroxidase Activity

Total peroxidase activity in plasma was measured following the procedure described by [67]. To do so, 15 µL of plasma in duplicate was diluted in 135 µL of HBSS without Ca^2+^ and Mg^2+^ in flat bottomed 96-well plates. Then, 50 µL of 20 mM 3,3′,5,5′-tetramethylbenzidine dihydrochloride (TMB; Sigma, St. Louis, MO, USA) and 50 µL of 5 mM hydrogen peroxide was added, resulting in a change in colour of the mixture that turned blue. The colour change reaction was stopped after 2 min by adding 50 µL of 2 M sulphuric acid and the optical density was read at 450 nm in a Synergy HT microplate reader, Biotek. Two wells with 150 µL of HBSS were used as blanks. The peroxidase activity (units/mL plasma) was determined defining one unit of peroxidase as that which produces an absorbance change of 1 optical density (OD).

#### 4.5.2. Antiprotease Activity

The method described by Ellis [68] was modified and adapted for 96-well microplates [35]. Firstly, 10 µL of plasma was incubated with the same volume of trypsin solution (5 mg/mL in NaHCO_3_ 5 mg/mL, pH 8.3) for 10 min at 22 °C in polystyrene microtubes. Afterwards, 100 µL of phosphate buffer (NaH_2_PO_4_, 13.9 mg/mL, pH 7.0) and 125 µL of azocasein (20 mg/mL in NaHCO_3_, 5 mg/mL, pH 8.3) were added and the mixture was incubated for 1 h at 22 °C. Then, 250 µL of trichloroacetic acid was added to the microtubes and incubated for 30 min at 22 °C. Finally, the mixture was centrifuged at 10,000× *g* for 5 min at room temperature and 100 µL of supernatants were transferred to a 96-well plate in duplicate containing 100 µL of 1N NaOH. One blank of phosphate buffer saline only was used in the protocol, and the reference sample was obtained using phosphate-buffered saline instead of plasma. The percentage of trypsin activity was calculated as follows:

% non-inhibited trypsin = (Sample absorbance × 100)/Reference sample

% inhibited trypsin = 100 − % non-inhibited trypsin

#### 4.5.3. Protease Activity

For the evaluation of this parameter, all procedures followed the same order and quantities of antiprotease activity protocol, except that the first step did not include the incubation with trypsin and the period of incubation with phosphate buffer and azocasein was maintained in constant agitation for 24 h.

### 4.6. Oxidative Stress

#### 4.6.1. Liver Homogenization

Liver tissues were homogenized with 10 volumes of ultrapure water, using a pellet mixer. A 200 µL aliquot was separated into a microtube with 4 µL BHT (2,6-Di-tert-butyl-4-methylphenol) 4% with methanol for lipid peroxidation (LPO) evaluation. One volume of tissue homogenate was mixed with one volume of potassium phosphate buffer (0.2 M, pH 7.4) and centrifuged at 10,000× *g* and 4 °C, for 20 min. The supernatants were stored at −80 °C until antioxidant enzymes activities were analysed.

#### 4.6.2. Lipid Peroxidation

LPO was determined following the method described by Bird and Draper [69]. In brief, 100 μL of cold 100% trichloroacetic acid was added to each sample and vortex. Then, 1 mL of 0.73% 2-thiobarbituric acid, Tris-HCl (60 mM) and 0.1 mM DTPA (pH 7.4) solution was added to each sample and blanks and vortex. After that, the microtubes were incubated for 1 h at 100 °C in a laboratory oven and centrifuged for 5 min at 15,000× *g*. Finally, the 200 µL of supernatant was transferred to a microplate in triplicates. The absorbance was measured at 535 nm and LPO was expressed as nmol of thiobarbituric acid reactive substances (TBARS) formed per g of wet tissue.

#### 4.6.3. Catalase Activity

Catalase (CAT) activity was measured following the consumption of the substrate (H_2_O_2_) seen by a decrease in absorbance at 240 nm, as described by Clairborne [70], and adapted to a microplate. In a microplate suited for UV light, triplicates of 10 µL of sample diluted to 0.7 mg/mL of protein were added, along with 140 µL of potassium phosphate (0.05 M, pH 7) and 150 µL of 30% H_2_O_2_. The absorbance was read at 240 nm for 2 min and catalase activity was expressed in U per mg of protein, using the H_2_O_2_ molar extinction coefficient at 240 nm of 40 M/cm.

#### 4.6.4. Superoxide Dismutase Activity

Superoxide dismutase (SOD) activity was monitored according to Almeida et al. [71], using the cytochrome C method, with xanthine/xanthine oxidase as the source of superoxide radicals. A reaction solution containing 50 mM potassium phosphate buffer (pH 7.8; and 1 mM Na-EDTA), 0.7 mM xanthine, 0.03 mM cytochrome C, 0.1 mM Na-EDTA and 0.03 U/mL xanthine oxidase was added to the sample triplicates diluted to 0.3 mg/mL of protein. Activity is reported in units of SOD per mg of protein. One unit of activity was defined as the amount of enzyme necessary to produce 50% inhibition of the cytochrome C reduction rate.

#### 4.6.5. Glutathione-S-Transferase Activity

Glutathione-S-transferase (GST) activity was determined following the method of Habig et al. [72] adapted to microplate by Frasco and Guilhermino [73]. The reaction mixture included 0.2 M potassium phosphate buffer (pH 6.5), 10 mM reduced glutathione (GSH), 60 mM 1-chloro-2,4-dinitrobenzene (CDNB) and triplicates of sample diluted to 0.7 mg/mL of protein. Absorbance was recorded at 340 nm for 5 min with 20 s intervals. GST activity was expressed as mU per mg of protein, using the molar extinction coefficient at 340 nm of 9.6 × 10^6^ M/cm.

#### 4.6.6. Total Glutathione

Total glutathione (tGSH) was determined as the rate of TNB^2−^ formation with an extinction coefficient of DTNB chromophore formed of 14.1 × 10^3^ M/cm [74,75].

#### 4.6.7. Protein Concentration

Protein concentration was measured using the Pierce™ BCA Protein Assay Kit, with bovine serum albumin as the standard, according to the manufacturer’s instructions.

### 4.7. Gene Expression Analysis

The extraction of the head kidney RNA was performed with NZY total RNA isolation kit (NZYTech, Lisbon, Portugal), following the manufacturer’s instructions. After extraction, RNA samples were quantified, and purity was assessed by spectrophotometry using DeNovix DS-11 FX (Wilmington, DE, USA).

NZY first-strand cDNA synthesis kit (NZYTech, Lisbon) was used for transcription of the obtained RNA to cDNA. This step also allowed us to standardize our samples (50 ng/µL of cDNA) on a final volume of 20 µL. Reverse transcriptase was then performed on Veriti DX 96-well Thermal Cycler (Applied Biosystems, Foster City, CA, USA).

Real-time quantitative PCR was carried out in duplicate for each reaction with the CFX384 Touch Real-Time PCR Detection System (Biorad, Hercules, CA, USA), with 15 genes (Table 1) being selected and studied according to their influence on the immune answer. Primer efficiency was tested for each gene with results varying between 116 and 87%. Cycling conditions were identical among different genes, varying only on the annealing temperature, consisting of 10 min at 95 °C for initial denaturation, followed by 40 cycles of 95 °C for 15 and primer annealing temperature for each gene for 1 min.

For each target, gene samples were normalized using EF-1α gene as housekeeping, and subsequently the Pfaffl method [76] was used for gene expression calculations.

### 4.8. Statistical Analysis

For all parameters analysed, the mean and standard deviation were calculated for each treatment and time group. Data were analysed for normality and homogeneity of variance and Log transformed before statistical treatment when needed. Data were analysed using *t*-Student’s test between all combinations of different groups. The performance of statistical analyses occurred under SPSS 26 program for WINDOWS. The level of significance used was *p* ≤ 0.05 for all statistical tests.

## Figures and Tables

**Table 1:** Immune-related genes analysed by real-time PCR.

Gene	Acronym	Accession Number	Annealing Temperature (°C)	Amplicon Length (bp)	Primer Sequence (5′-3′)
Elongation Factor 1α	EF-1α	AF184170	58	87	F: CTGTCAAGGAAATCCGTCGT R:TGACCTGAGCGTTGAAGTTG
Heat-Shock Protein 70	HSP70	DQ524995.1	55	124	F: ACGGCATCTTTGAGGTGAAG R:TGGCTGATGTCCTTCTTGTG
Non-specific cytotoxic cell receptor protein	NCCRP	AY651258.1	60	100	F: ACTTCCTGCACCGACTCAAG R:TAGGAGCTGGTTTTGGTTGG
Interleukin 34	IL-34	JX976629.1	60	214	F: CATCAGGGTTCATCACAACG R: GACTCCCTCTGCATCCTTGA
Hepcidin	HEP	EF625901	60	382	F: GCCATCGTGCTCACCTTTAT R:CCTGCTGCCATACCCCATCTT
Major histocompatibility complex I	MHC I	DQ211541.1	60	104	F: CGATGGAACCTTCCAGATGA R:CCTCGTTCACACCAGAGAGC
Major histocompatibility complex II γ	MHC II	AM920665.1	60	107	F: ACAACATGAACGCTGAGCTG R:CTCGTCCACAGAGTCATCCA
Interleukin 1 β	IL-1β	AJ277166.2	60	245	F: TCTTCAAATTCCTGCCACCA R:CAATGCCACCTTGTGGTGAT
Colony stimulating factor-1 receptor	CSF1R	AM050293	60	129	F: ACGTCTGGTCCTATGGCATC R:AGTCTGGTTGGGACATCTGG
Transforming growth factor β1	TGF-β1	AF424703.1	58	132	F: TCTGGGGTGGAAATGGATAC R: CTCCTGGGTTGTGATGCTTA
Caspase 1	CASP-1	AM490060	59	92	F: ACGAGGTGGTGAAACACACA R: GTCCGTCTCTTCGAGTTTCG
β-Defensin	β-DEF	FM158209	60	101	F: CCCCAGTCTGAGTGGAGTGT R: AATGAGACACGCAGCACAAG
Interleukin 10	IL-10	JX976626	57	65	F: AACATCCTGGGCTTCTATCTG R: GTGTCCTCCGTCTCATCTG
Transferrin	TRANSF	JF309047	60	100	F: CAGGACCAGCAGACCAAGTT R: TGGTGGAGTCCTTGAAGAGG
Haptoglobin	HAPT	KU940258	60	120	F: TTCCTCTTACTTGCCCTGGA R: CAGGGCCTGAAGCTCTACTG

## Data Availability

All data is provided in the main text or supplementary files.

## Supplementary Materials

The following supporting information can be downloaded at: https://www.mdpi.com/article/10.3390/ijms23031561/s1.
