# Supplementary material for: Short-Term Immune Responses of Gilthead Seabream (Sparus aurata) Juveniles against Photobacterium damselae subsp. piscicida
Source: Int J Mol Sci. 2022 Jan 29;23(3):1561. doi: 10.3390/ijms23031561 (PMC8836189; doi:10.3390/ijms23031561)
Supplement: Supplementary file 1 [file ijms-23-01561-s001.zip › ijms-1568589-supplementary.pdf]

Table S1. White blood cells (WBC, x 10^4), red blood cells (RBC, x 10^6), heamatocrit (Ht, %), haemoglobin (Hg, g/dL), mean corpuscular volume (MCV, μm^3), mean corpuscular heamoglobin (MCH, pg/cell) and mean corpuscular heamoglobin concentration (MCHC, g/100 mL) of gilthead seabream before and after bacterial or placebo challenge.

	0 h	3 h		6 h		9 h		24 h		48 h	
	**Control**	**Placebo**	**Infected**	**Placebo**	**Infected**	**Placebo**	**Infected**	**Placebo**	**Infected**	**Placebo**	**Infected**
**WBC**	5.68 ± 1.18^{aAB}	5.05 ± 0.94^{AB}	4.62 ± 1.02^{ab}	4.92 ± 0.37$^{B\#}$	3.77 ± 0.51$^{b\$}$	5.65 ± 0.60^{A}	4.10 ± 2.40^{ab}	4.47 ± 1.21^{AB}	5.18 ± 1.64^{ab}	5.42 ± 0.88^{AB}	5.05 ± 1.44^{ab}
**RBC**	1.85 ± 0.41^{aA}	1.82 ± 0.28^{A}	1.58 ± 0.44^{abc}	1.51 ± 0.38^{AB}	1.44 ± 0.38^{abc}	1.38 ± 0.28^{B}	1.18 ± 0.24^{c}	1.45 ± 0.31^{AB}	1.54 ± 0.16^{b}	1.84 ± 0.50$^{AB\#}$	1.25 ± 0.14$^{c\$}$
**Ht**	26.80 ± 4.85^{abAB}	30.33 ± 3.61^{A}	27.60 ± 1.52^{a}	27.60 ± 2.88^{AB}	27.25 ± 4.99^{ab}	30.40 ± 4.39$^{AB\#}$	22.80 ± 2.68$^{bc\$}$	26.00 ± 2.68^{B}	22.00 ± 5.48^{abc}	31.17 ± 3.13$^{A\#}$	19.00 ± 2.71$^{c\$}$
**Hg**	1.37 ± 0.34^{bB}	1.86 ± 0.31^{A}	1.74 ± 0.23^{a}	1.30 ± 0.34^{BC}	1.64 ± 0.46^{ab}	1.59 ± 0.50^{AB}	1.17 ± 0.34^{b}	0.99 ± 0.27$^{C\$}$	1.54 ± 0.29$^{ab\#}$	1.31 ± 0.37$^{BC\#}$	0.74 ± 0.15$^{c\$}$
**MCV**	138.99 ± 25.25^{bB}	170.61 ± 35.32^{AB}	162.78 ± 21.70^{ab}	188.76 ± 25.83^{A}	166.24 ± 19.93^{ab}	194.98 ± 40.80^{A}	178.21 ± 30.49^{a}	184.78 ± 37.34^{A}	143.22 ± 32.13^{ab}	161.37 ± 36.98^{AB}	151.84 ± 7.39^{ab}
**MCH**	3.71 ± 1.23^{bcB}	5.48 ± 1.01^{A}	5.81 ± 1.31^{a}	4.57 ± 1.71^{AB}	5.12 ± 1.21^{ab}	5.83 ± 1.69^{A}	5.01 ± 1.13^{ab}	3.45 ± 0.68$^{B\$}$	5.05 ± 0.93$^{a\#}$	3.79 ± 1.45^{B}	3.04 ± 0.82^{c}
**MCHC**	2.78 ± 0.88^{abAB}	3.33 ± 0.94^{A}	3.33 ± 0.32^{a}	2.37 ± 0.69$^{AB\$}$	3.48 ± 0.55$^{a\#}$	2.66 ± 0.81^{AB}	2.69 ± 1.01^{ab}	1.92 ± 0.55$^{B\$}$	3.20 ± 0.83$^{ab\#}$	2.08 ± 0.44^{B}	2.23 ± 0.49^{b}

Data are expressed as means ± SD (n=12 for control animals and n=6 on time course animals). Different lower case letters stand for significant differences between times regarding control and infected groups, while symbol stands for differences between treatments on the same sampling time. Different capital letters indicate differences between times within control and placebo groups. (*t*-student test or Kruskal-Wallis; $P \leq 0.05$).

Table S2. Absolute values (x 10^4) of peripheral blood leukocytes (neutrophils, monocytes, lymphocytes and thrombocytes) of gilthead seabream before and after bacterial or placebo challenge.

	0 h	3 h		6 h		9 h		24 h		48 h	
	**Control**	**Placebo**	**Infected**	**Placebo**	**Infected**	**Placebo**	**Infected**	**Placebo**	**Infected**	**Placebo**	**Infected**
**Neutrophils**	0.34 ± 0.26^{bC}	0.93 ± 0.50^{AB}	1.28 ± 0.72^{a}	1.22 ± 0.25^{A}	1.48 ± 0.49^{a}	0.81 ± 0.44^{AB}	1.03 ± 0.31^{a}	0.61 ± 0.28^{BC}	1.29 ± 0.93^{a}	0.49 ± 0.20^{BC}	1.01 ± 0.80^{a}
**Monocytes**	0.28 ± 0.10^{bc}	0.40 ± 0.31	0.37 ± 0.24^{abc}	0.30 ± 0.10	0.20 ± 0.09^{c}	0.37 ± 0.16	0.10 ± 0.03^{d}	0.22 ± 0.13	0.45 ± 0.21^{a}	0.33 ± 0.13	0.51 ± 0.26^{ab}
**Lymphocytes**	1.44 ± 0.28^{aA}	0.98 ± 0.35^{B}	0.82 ± 0.21^{bc}	0.94 ± 0.34$^{B\#}$	0.50 ± 0.15$^{d\$}$	1.21 ± 0.27$^{AB\#}$	0.61 ± 0.22$^{cd\$}$	1.06 ± 0.21^{B}	1.00 ± 0.26^{b}	1.33 ± 0.31$^{AB\#}$	0.81 ± 0.18$^{bc\$}$
**Thrombocytes**	3.40 ± 0.74^{aA}	2.74 ± 0.72^{AB}	2.14 ± 0.62^{bc}	2.45 ± 0.40$^{B\#}$	1.59 ± 0.30$^{c\$}$	3.26 ± 0.25$^{A\#}$	1.77 ± 0.91$^{bc\$}$	2.58 ± 0.81^{AB}	2.45 ± 0.51^{b}	3.26 ± 0.71^{A}	2.58 ± 0.61^{b}

Data are expressed as means ± SD (n=12 for control animals and n=6 on time course animals). Different lower case letters stand for significant differences between times regarding control and infected groups, while symbol stands for differences between treatments on the same sampling time. Different capital letters indicate differences between times within control and placebo groups. (*t*-student test or Kruskal-Wallis; $P \leq 0.05$).

Table S3. Plasma antiprotease (%), peroxidase (units/mL) and proteases activities (%) of gilthead seabream before and after bacterial or placebo challenge.

	0 h	3 h		6 h		9 h		24 h		48 h	
	**Control**	**Placebo**	**Infected**	**Placebo**	**Infected**	**Placebo**	**Infected**	**Placebo**	**Infected**	**Placebo**	**Infected**
**Antiprotease activity**	85.36 ± 5.70^{cB}	88.22 ± 4.71^{AB}	89.04 ± 4.11^{bc}	87.63 ± 6.72^{AB}	84.71 ± 7.07^{bc}	84.85 ± 5.60^{B}	87.30 ± 5.01^{bc}	87.00 ± 4.71^{AB}	91.45 ± 4.14^{ab}	91.42 ± 2.48$^{A\$}$	95.49 ± 1.65$^{a\#}$
**Peroxidase activity**	20.81 ± 9.54^{A}	24.80 ± 5.25^{A}	22.54 ± 8.37	12.01 ± 2.88$^{B\$}$	23.30 ± 9.24$^{\#}$	20.00 ± 6.16^{A}	21.39 ± 12.60	18.04 ± 5.28^{AB}	33.02 ± 15.63	32.57 ± 18.66^{AB}	21.46 ± 9.77
**Protease activity**	74.43 ± 25.10	82.16 ± 7.09	73.85 ± 20.60	82.10 ± 12.75	66.17 ± 30.93	71.36 ± 26.09	66.76 ± 38.88	67.29 ± 16.79	67.14 ± 8.75	60.63 ± 20.81	59.45 ± 15.67

Data are expressed as means ± SD (n=12 for control animals and n=6 on time course animals). Different lower case letters stand for significant differences between times regarding control and infected groups, while symbol stands for differences between treatments on the same sampling time. Different capital letters indicate differences between times within control and placebo groups. (*t*-student test or Kruskal-Wallis; $P \leq 0.05$).

Table S4. Liver lipid peroxidation (LPO, TBARS/g), Total glutathione (tGSH, nmol/mg protein), Glutathione S-transferase activity (GST, mU/mg protein), Catalase activity (CAT, U/mg protein) and Superoxide dismutase activity (SOD, U/mg protein) of gilthead seabream before and after bacterial or placebo challenge.

	0 h	3 h		6 h		9 h		24 h		48 h	
	**Control**	**Placebo**	**Infected**	**Placebo**	**Infected**	**Placebo**	**Infected**	**Placebo**	**Infected**	**Placebo**	**Infected**
**LPO activity**	37.31 ± 4.25^{BCc}	37.23 ± 4.23^{BC}	37.75 ± 5.13^{bc}	40.66 ± 4.18^{AB}	42.85 ± 9.90^{abc}	44.34 ± 4.63^{A}	46.55 ± 4.35^{a}	45.87 ± 5.25^{A}	44.41 ± 7.52^{ab}	34.31 ± 4.29$^{C\$}$	42.46 ± 3.12$^{ab\#}$
**tGSH activity**	51.63 ± 24.23^{Ba}	87.54 ± 32.64^{A}	74.59 ± 35.96^{a}	51.57 ± 25.03^{AB}	46.97 ± 20.98^{a}	44.03 ± 29.90^{B}	44.02 ± 31.33^{ab}	67.09 ± 22.99$^{AB\#}$	8.12 ± 6.91$^{b\$}$	57.24 ± 20.72^{AB}	35.41 ± 24.76^{ab}
**GST activity**	144.12 ± 48.43^{cC}	212.97 ± 89.47^{AB}	182.24 ± 79.42^{abc}	148.69 ± 34.04^{BC}	239.46 ± 32.87^{a}	153.45 ± 72.77^{ABC}	251.49 ± 62.12^{a}	175.06 ± 87.30^{ABC}	221.06 ± 75.25^{ab}	208.25 ± 23.99^{A}	150.13 ± 40.50^{bc}
**CAT activity**	11.69 ± 1.57^{Bb}	12.99 ± 3.15^{B}	14.11 ± 4.04^{abc}	15.54 ± 5.78^{AB}	13.52 ± 3.42^{ab}	11.58 ± 0.86$^{B\#}$	8.53 ± 0.84$^{c\$}$	11.66 ± 3.63$^{B\$}$	16.81 ± 2.18$^{a\#}$	16.64 ± 1.55^{A}	16.51 ± 2.06^{a}
**SOD activity**	8.29 ± 4.36^{ABab}	20.69 ± 19.44^{A}	10.24 ± 12.08^{ab}	10.42 ± 14.73^{AB}	14.04 ± 10.19^{a}	3.64 ± 1.87^{B}	11.83 ± 8.17^{ab}	13.20 ± 4.90^{A}	14.98 ± 13.98^{a}	12.44 ± 11.09^{AB}	3.70 ± 0.68^{b}

Data are expressed as means ± SD (n=12 for control animals and n=6 on time course animals). Different lower case letters stand for significant differences between times regarding control and infected groups, while symbol stands for differences between treatments on the same sampling time. Different capital letters indicate differences between times within control and placebo groups. (*t*-student test or Kruskal-Wallis; $P \leq 0.05$).

Table S5. Quantitative expression of heat shok protein 70 (*hsp70*), non-specific citotoxic cell receptor protein 1 (*nccrp1*), interleukin 34 (*il-34*), hepcidin (*hep*), major histocompatibility complex I (*mhc I*), major histocompatibility complex II (*mhc II*), interleukinβ1 (*il-1β*), colony stimulation factor 1 receptor (*csf1r*), transforming growth factorβ1 (*tgf- β1*), caspase 1 (*casp1*), β-defensin (*β-def*), interleukin 10 (*il-10*), haptoglobin (*hptg*) and transferrin (*transf*) in the head kidney of gilthead seabream juveniles before and after bacterial or placebo challenge.

	**0 h**	**3 h**		**6 h**		**9 h**		**24 h**		**48 h**	
	**Control**	**Placebo**	**Infected**	**Placebo**	**Infected**	**Placebo**	**Infected**	**Placebo**	**Infected**	**Placebo**	**Infected**
hsp70	0.91 ± 0.30^{cC}	1.64 ± 0.58^{AB}	2.08 ± 2.17^{abc}	3.46 ± 4.55^{A}	6.23 ± 9.10^{ab}	2.75 ± 1.74^{A}	2.13 ± 1.61^{ab}	0.96 ± 0.49$^{BC\$}$	8.64 ± 14.72$^{a\#}$	5.46 ± 7.54^{AB}	1.79 ± 1.85^{bc}
nccrp	1.57 ± 0.61^{bA}	1.67 ± 0.39^{A}	1.48 ± 0.61^{bc}	1.79 ± 0.67$^{A\#}$	0.83 ± 0.46$^{c\$}$	2.34 ± 0.85$^{A\#}$	0.87 ± 0.42$^{c\$}$	1.73 ± 1.14^{AB}	1.90 ± 0.42^{ab}	0.87 ± 0.20$^{B\$}$	2.62 ± 0.88$^{a\#}$
il-34	1.12 ± 0.55^{cA}	1.22 ± 0.58^{A}	1.14 ± 0.60^{c}	0.71 ± 0.34^{AB}	1.23 ± 0.54^{c}	0.87 ± 0.21$^{AB\$}$	1.78 ± 0.44$^{b\#}$	0.62 ± 0.41$^{AB\$}$	3.19 ± 0.68$^{a\#}$	0.42 ± 0.25$^{B\$}$	2.00 ± 0.90$^{abc\#}$
hep	2.42 ± 1.41^{aA}	1.92 ± 0.92^{AB}	1.39 ± 0.87^{abc}	1.24 ± 0.49^{BC}	0.62 ± 0.58^{bcd}	1.54 ± 0.75^{ABC}	0.71 ± 0.55^{cd}	1.30 ± 0.59$^{ABC\#}$	0.36 ± 0.14$^{d\$}$	0.99 ± 0.37^{C}	1.35 ± 0.18^{b}
mhc-I	0.46 ± 0.19^{bC}	0.77 ± 0.24^{A}	0.86 ± 0.82^{ab}	1.11 ± 1.56^{BC}	2.50 ± 3.65^{ab}	1.01 ± 0.82^{AB}	0.98 ± 0.82^{a}	0.29 ± 0.10$^{C\$}$	3.57 ± 5.17$^{a\#}$	1.20 ± 1.81$^{BC\$}$	0.97 ± 0.26$^{a\#}$
mhc-II	0.73 ± 0.35^{AB}	0.98 ± 0.44^{A}	0.76 ± 0.46	0.65 ± 0.22^{ABC}	0.61 ± 0.39	0.68 ± 0.15^{AB}	0.78 ± 0.17	0.36 ± 0.19$^{\$C}$	0.95 ± 0.55$^{\#}$	0.46 ± 0.33^{BC}	0.78 ± 0.28
il-1β	1.06 ± 0.74^{bB}	4.87 ± 2.15$^{A\$}$	1074.06 ± 313.88$^{a\#}$	14,47 ± 22.50^{A}	120.94 ± 99.01^{a}	14.30 ± 15.89$^{AB\$}$	488.64 ± 264.46$^{a\#}$	2.03 ± 1.15$^{AB\$}$	993.44 ± 781.32$^{a\#}$	15.78 ± 28.89^{AB}	188.67 ± 189.32^{a}
csf1r	1.09 ± 0.51^{a}	0.87 ± 0.25$^{\#}$	0.53 ± 0.18$^{bc\$}$	0.65 ± 0.23	0.38 ± 0.18^{c}	0.85 ± 0.29	0.52 ± 0.30^{bc}	0.62 ± 0.34	1.26 ± 0.76^{ab}	0.62 ± 0.30$^{\$}$	1.18 ± 0.44$^{a\#}$
tgf-β1	0.93 ± 0.47^{ab}	0.88 ± 0.43	0.75 ± 0.32^{ab}	0.69 ± 0.25	0.95 ± 0.84^{ab}	0.89 ± 0.36	0.71 ± 0.27^{b}	0.69 ± 0.34	2.19 ± 2.40^{a}	0.52 ± 0.33$^{\$}$	1.19 ± 0.36$^{a\#}$
casp1	0.71 ± 0.21^{Bc}	1.39 ± 0.40^{A}	1.48 ± 1.45^{bc}	2.70 ± 3.92^{A}	4.84 ± 7.71^{abc}	1.64 ± 0.69^{A}	1.61 ± 1.37^{ab}	0.62 ± 0.18$^{B\$}$	7.05 ± 12.02$^{a\#}$	2.24 ± 3.37^{AB}	1.16 ± 0.39^{abc}
β-def	1.98 ± 3.30	0.82 ± 0.28	0.74 ± 0.74	0.49 ± 0.32	1.18 ± 1.25	1.25 ± 1.35	0.76 ± 0.40	1.39 ± 2.02	0.98 ± 0.37	14.80 ± 30.96	2.20 ± 1.14
il-10	0.99 ± 0.33^{b}	1.37 ± 0.43$^{\$}$	5.03 ± 3.07$^{a\#}$	2.59 ± 3.09	6.28 ± 7.77^{a}	2.13 ± 1.61$^{\$}$	6.04 ± 1.56$^{a\#}$	0.75 ± 0.48$^{\$}$	7.01 ± 9.44$^{a\#}$	2.57 ± 3.57	0.73 ± 0.31^{b}
hptg	0.81 ± 0.74^{bB}	2.18 ± 2.20^{AB}	12.20 ± 16.51^{a}	12.06 ± 18.05^{A}	59.31 ± 79.39^{a}	6.80 ± 6.91$^{A\$}$	146.31 ± 74.75$^{a\#}$	1.71 ± 1.95$^{AB\$}$	153.11 ± 122.06$^{a\#}$	14.06 ± 27.37^{AB}	22.23 ± 19.71^{a}
transf	3.35 ± 2.06^{c}	2.88 ± 1.86	19.43 ± 17.14^{abc}	4.57 ± 3.70	66.39 ± 54.37^{bc}	2.71 ± 1.12$^{\$}$	470.05 ± 252.82$^{ab\#}$	2.18 ± 1.19$^{\$}$	2038.86 ± 1305.47$^{a\#}$	7.47 ± 13.85$^{\$}$	1159.48 ± 982.11$^{ab\#}$

Data are expressed as means ± SD (n=12 for control animals and n=6 on time course animals). Values present the relative expression of target genes previously normalized to Elongation Factor 1α (ef-1α). Different lower case letters stand for significant differences between times regarding control and infected groups, while symbol stands for differences between treatments on the same sampling time. Different capital letters indicate differences between times within control and placebo groups. (*t*-student test or Kruskal-Wallis; $P \leq 0.05$).
